# An Image-Based Prior Knowledge-Free Approach for a Multi-Material Decomposition in Photon-Counting Computed Tomography

**DOI:** 10.3390/diagnostics14121262

**Published:** 2024-06-14

**Authors:** Jonas Neumann, Tristan Nowak, Bernhard Schmidt, Joachim von Zanthier

**Affiliations:** 1 Quantum Optics and Quantum Information Group (QOQI), Friedrich-Alexander-Universität Erlangen-Nürnberg, Staudtstr. 1, 91058 Erlangen, Germany; 2 Siemens Healthineers AG, Siemensstr. 3, 91301 Forchheim, Germany

**Keywords:** computed tomography, spectral ct, photon-counting ct, photon-counting detector, multi-material decomposition, k-edge imaging

## Abstract

Photon-counting CT systems generally allow for acquiring multiple spectral datasets and thus for decomposing CT images into multiple materials. We introduce a prior knowledge-free deterministic material decomposition approach for quantifying three material concentrations on a commercial photon-counting CT system based on a single CT scan. We acquired two phantom measurement series: one to calibrate and one to test the algorithm. For evaluation, we used an anthropomorphic abdominal phantom with inserts of either aqueous iodine solution, aqueous tungsten solution, or water. Material CT numbers were predicted based on a polynomial in the following parameters: Water-equivalent object diameter, object center-to-isocenter distance, voxel-to-isocenter distance, voxel-to-object center distance, and X-ray tube current. The material decomposition was performed as a generalized least-squares estimation. The algorithm provided material maps of iodine, tungsten, and water with average estimation errors of 4% in the contrast agent maps and 1% in the water map with respect to the material concentrations in the inserts. The contrast-to-noise ratio in the iodine and tungsten map was 36% and 16% compared to the noise-minimal threshold image. We were able to decompose four spectral images into iodine, tungsten, and water.

## 1. Introduction

Two decades after the advent of spectral computed tomography (CT) modalities [1,2,3,4,5,6] and the introduction of material-selective algorithms [7,8,9,10,11,12,13,14,15], energy-discriminating photon-counting detector (PCD) CT systems have become commercially available [16,17]. Analogous to established dual-energy techniques on energy-integrating detectors, such as dual-source, kV-switching, or the use of dual-layer detectors, threshold image acquisition with PCDs allows two-material decomposition of the input images into a pair of base materials. Among others, this enables the reconstruction of virtual non-contrast, iodine and virtual monoenergetic images [18,19].

PCD CT systems, for the first time, enable the acquisition of more than two spectral images in a single scan and thus the decomposition into more than two base materials, hereafter called multi-material decomposition (MMD). In combination with the development of novel contrast agents (CAs) based on materials with high atomic number and thus high K-absorption edges (hereafter called K-edge CAs), this opens up new scanning techniques [20,21,22]. As an example, a patient with a known history of hemochromatosis could be injected with a K-edge CA, which would allow an accurate iron quantification via MMD into iron, liver tissue, and the K-edge CA. To examine the lung ventilation, a patient could breathe in xenon and be injected with a K-edge CA to allow MMD into xenon, the K-edge CA, and water, which indicates soft tissue.

CT-based MMD has already been investigated [21,23,24,25,26,27,28,29,30,31,32], including in prototype CT systems and simulations which provided at least three spectral datasets. It was also assessed in clinically used CT systems, which provided at most two spectral datasets. Consequently, the clinical system required repeated measurements or boundary conditions resulting from prior knowledge of the scanned object or assumptions as volume conservation or mass conservation for instance [23,33].

The new commercial CT systems involving PCD overcome these limitations. These systems allow a one-shot acquisition of four spectral images and thus prior knowledge-free MMD by a single scan. However, PCD CT systems with challenging effects such as pile-up and charge sharing require calibration of material CT numbers for an accurate material quantification [16,34].

In this work, we present and test an image-based prior knowledge-free method to perform MMD on the new PCD CT systems involving models to calibrate material CT numbers. We first describe the general method, applicable to a set of arbitrary materials with distinct spectral behavior and an equal or larger set of spectral images. To demonstrate the method’s feasibility, we applied the approach to scans of an abdominal phantom using different phantom diameters, tube currents, and positions. Our material system included iodine, tungsten, and water. We acquired four spectral images per scan, which are energy threshold images in the used CT system.

We investigated the deviations of the material concentration estimation (CE) from the true value and the statistical uncertainty of the MMD results, analyzed by comparing the contrast-to-noise ratio (CNR) in the algorithm input and output.

In contrast to previous works [23,24,25,26,27,28,29,30,31,32,33,35,36], we used commercial hardware allowing one-shot acquisition of more than two spectral datasets. This is the first time a prior knowledge-free calibration and decomposition approach involving more than two materials was tested on a clinical PCD CT system using a four-threshold image acquisition protocol. In the literature, we have not found similarly detailed models as those we used to calibrate material CT numbers. We moreover created a model for the image noise covariance matrix (INCM) to reduce the random noise in the material maps.

## 2. Materials and Methods

**Figure 1:**
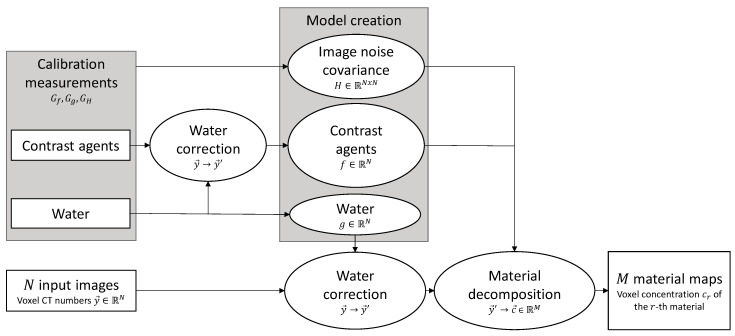
Summary of the general approach of obtaining M material maps from N spectral input images. Model creation: the covariance of the spectral images is measured to model the image noise covariance matrix. Averaged CT numbers of contrast agent-filled image regions are corrected by those of water before modeling. A model of the water CT numbers is built separately. Decomposition: given N input images, each individual voxel is corrected using the water model and then fed into the decomposition algorithm, whose output translates into material concentrations.

Our approach is introduced in Section 2.1. We first present the method structure, outlined in Figure 1, and then discuss the method details involving model functions for material CT numbers and the INCM. All models are created analytically and do not require manually fine-tuned initial conditions to converge to a good optimum. The proposed model functions are further discussed in Appendix A.

Two measurement series, one to calibrate the models and one to test the calibrated models, will be described in Section 2.2. We finally discuss the analysis of the precision and accuracy of the material images resulting from applying our approach to the test data as described in Section 2.3. The customized code for model creation, material decomposition, and image analysis was developed in MATLAB (Version R2021b, The MathWorks Inc., Natick, MA, USA).

### 2.1. General Method

The general method has two parts: First, the creation of CT number models of all materials of interest in the N spectral images series provided by the CT system and of a model of the INCM. Second, the decomposition of N input images into M≤N materials, called base materials (BMs), resulting in a vector $\vec{c}\in R^{M}$ of estimated BM concentrations for each image voxel.

- $\vec{y}\in R^{N}$ is the vector of CT numbers of one image voxel at fixed row and column in the different spectral images;
- $\vec{x}=(x_{1},\;x_{2},\ldots )$ are the parameters on which the models depend;
- $f=f(\vec{x};\vec{\alpha })$ is the model of a CA CT number in one spectral image with coefficients $\vec{\alpha }$ to calibrate and a set of ground truth data $G_{f}$;
- $g=g(\vec{x};\vec{\alpha })$ is the model of the water CT number in one spectral image with coefficients $\vec{\alpha }$ to calibrate and a set of ground truth data $G_{g}$;
- $H=H(\vec{x};\vec{\alpha })$ is the model of the INCM with coefficients $\vec{\alpha }$ to calibrate and a set of ground truth data $G_{H}$.

For each spectral image, a model of the CT numbers is created for the BMs and water even if water is not among the BMs as discussed in Section 2.1.2. Calibration measurements are performed to find ground truth at different parameters $\vec{x}$ for each model. Having acquired the ground truth, the user calibrates H based on $G_{H}$, g for each spectral image based on $G_{g}$, and f for each remaining BM and each spectral image based on $G_{f}$ and $G_{g}$, as discussed in Section 2.1.2 and Section 2.1.3.

Given the calibrated models and N spectral images to decompose, the material decomposition for a voxel with parameters $\vec{x}$ and CT numbers $\vec{y}$ is prepared as follows:

1. Design matrix $S\in R^{N\times M}$: If the r-th BM (r=1, …, M) is a CA, $S_{pr}$ is set to $f(\vec{x};\vec{\alpha })$ with f the model of the r-th BM CT number in the p-th spectral image (p=1, …, N), scaled to give the expected CT numbers at a concentration of 1 mg/mL for easy interpretation of the decomposition results. If the r-th BM is water, $S_{pr}$ is set to 1 for all *p* to ensure a linear water CE in the true water concentration for voxels containing at most water, interpreted as explained at the end of this section.
2. Observation vector ${\vec{y}}^{\prime }\in R^{N}$: For all p (p=1, …, N) and the corresponding water model g,
  (1) $$y_{k}^{\prime }=\frac{y_{k}-g\left(\vec{x};\vec{\alpha }\right)}{1+g\left(\vec{x};\vec{\alpha }\right)/(1000\;\mathrm{HU})}.$$
3. Covariance matrix $V\in R^{N\times N}$: V is set to $H(\vec{x};\vec{\alpha })$.
4. The user then estimates the material coefficients $\vec{\rho }\in R^{M}$ using the method of generalized LS. This means solving the linear system of equations
  (2) $$S^{T}V^{-1}S\vec{\rho }=S^{T}V^{-1}{\vec{y}}^{\prime }.$$
5. The result $\vec{\rho }$ is the best linear unbiased estimator for the overdetermined system
  (3) $$S\vec{\rho }={\vec{y}}^{\prime }.$$

‘Best’ means that among all linear functions in ${\vec{y}}^{\prime }$ which minimize the distance between $S\vec{\rho }$ and ${\vec{y}}^{\prime }$, the solution of Equation (2) has the lowest variance. Note that in the case of M=N, Equation (2) is identical to Equation (3) and can be solved without LS methods and the INCM. Furthermore, in the case of M<N, the solution of Equation (2) has a lower variance than the ordinary LS estimator ${\left(S^{T}S\right)}^{-1}S^{T}{\vec{y}}^{\prime }$.

The translation of $\vec{\rho }$ into $\vec{c}$ is as follows: if the r-th material is a CA, $\rho_{r}$ equals $c_{r}$ in units of mg/mL. Note that the user could fill the r-th column in S with the expected CT numbers at any non-zero concentration q mg/mL. The interpretation is then that $\rho_{r}\cdot q$ equals $c_{r}$ in units of mg/mL.

In the case of water, $c_{r}={(1000+\rho }_{r})\;\mathrm{mg}/\mathrm{mL}$, which means that $\rho_{r}=0$ corresponds to a water-filled image voxel plus possible CA and $\rho_{r}=-1000$ means water absence.

By repeating this decomposition for all image voxels of the N input images, M material maps displaying material concentrations are generated.

Note that the choice of distinguishable materials is limited by their spectral separation. Since in CT, the absorption behavior of human body materials is dominated by the photoelectric effect and Compton scattering [34], discrimination of more than two materials requires all further materials to exhibit a unique K-edge absorption in the diagnostic energy window.

#### 2.1.1. Model Parameters

All our models used an individual subset of the following calibration parameters:

- Object water-equivalent diameter (WED) [mm] ($x_{1}$);
- X-ray tube current [mA] ($x_{2}$);
- Voxel distance to the isocenter [mm] ($x_{3}$);
- Voxel distance to the object center [mm] ($x_{4}$);
- Object off-centering [mm] ($x_{5}$).

**Figure 2:**
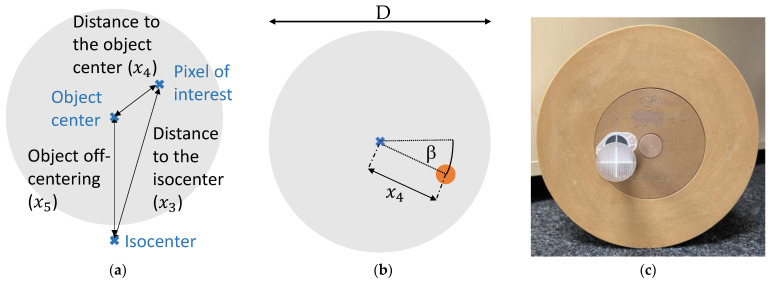
Object geometry. (**a**) Definition of the distance measures. (**b**) Exemplary evaluated circular region defined by its center’s distance $x_{4}$ to the phantom center and the angle β to the horizontal line through the phantom center. (**c**) Calibration phantom for the exemplary case of D=200 mm. We evaluated a circular region at $x_{4}=30\;\mathrm{mm}$ and β=180°, which is in the drill hole filled by one of the inserts. Moreover, at several angles β (−90°,−45°, 0°,+45°,+90°) and distances $x_{4}$ to phantom center, a circular region in WEP was evaluated. For each scanned phantom diameter D, we used $x_{4}=30\;\mathrm{mm}$ and at D≥200 mm and D≥300 mm, we additionally included $x_{4}=70\;\mathrm{mm}$ and 120 mm, respectively.

The WED is slice-wise computed from the image CT numbers. It equals the diameter of a disk of water providing the same area-integrated attenuation as the measured object [37]. Each image voxel has associated distance measures $x_{3}{-x}_{5}$ visualized in Figure 2a. The object off-centering is the distance between the isocenter and the center of mass of the measured object.

#### 2.1.2. Material Model Creation

**Figure 3:**
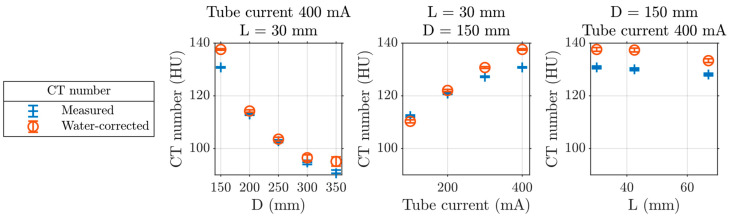
CT number of iodine solution (c=10 mg/mL) positioned in a water-equivalent plastic cylinder of diameter D in the image using all photons above 90 keV of an energy-discriminating photon-counting detector CT system at 140 kV tube voltage and after water correction, described in Section 2.1.2, with fixed parameters as shown. L denotes the distance of the solution to the isocenter. The error bars indicate the standard error on the iodine solution region mean (averaged over 1.6 mL) of the individual points due to quantum noise.

The material models consider several parameters which affect the measured CT number in a PCD CT system exemplarily shown in Figure 3. With an increasing object diameter, the X-ray spectrum becomes harder. This leads to a change in CA attenuation and hence the measured CT number. The PCD’s sensitivity to the photon flux due to pile-up and charge sharing explains the non-zero correlation between CT number and tube current and between CT number and distance to the isocenter due to form filters on top of the X-ray source.

To calibrate the models to different parameters, the user performs calibration scans with different parameter configurations $C=\left\{{\vec{x}}^{1},\;\ldots ,\;{\vec{x}}^{K}\right\}$ with K equal to or larger than the number of coefficients $\vec{\alpha }$ of the model function $f(\vec{x};\vec{\alpha })$, for each spectral image and CA in the BMs. This leads to a set of ground truth values $G_{f}=\{({\vec{x}}^{k};y_{k})\;:k=1,\ldots ,\;K\}$ of the CT numbers in units of HU. C must be a subset of the parameter configurations used to acquire $G_{g}$.

We recommend modeling CA CT numbers in a spectral image defined by the following function:

(4) $$f\left(\vec{x};\vec{\alpha }\right)=\alpha_{1}+\left(\sum_{i=1}^{3}\alpha_{i+1}x_{i}+\alpha_{i+4}{x_{i}}^{2}\right)+\alpha_{8}x_{1}x_{2}+\alpha_{9}x_{1}x_{3}+\alpha_{10}x_{2}x_{3}$$

with constant numbers $\left\{\alpha_{i}\in R:i=1,\;\ldots ,\;10\right\}$ left to be determined using $G_{f}$. If water-filled regions do not all receive a CT number of 0 HU from the CT system, an intermediate computation, called *water correction*, is required. This consists of replacing the ground truth CT number $y_{k}$ by $y_{k}^{\prime }$ with

(5) $$y_{k}^{\prime }=\frac{y_{k}-w}{1+w/(1000\;\mathrm{HU})}$$

for each k=1,…, K where w is the CT number of water measured at the same configuration ${\vec{x}}^{k}$ [38]. According to the Hounsfield scale which is used for image-based material decomposition, water-filled regions should receive a CT number of 0 HU from the CT system. Regions with aqueous CA solutions should receive a CT number that is proportional to their concentration, at least in the thin-absorber limit [39]. This is not everywhere the case in the images of the system we used. Applying Equation (5), we come closer to the preconditions while leaving material-free regions at −1000 HU.

Optimal model coefficients are then determined through an ordinary LS fit using the chosen model function f and the ground truth data $\left\{({\vec{x}}^{k};y_{k}^{\prime })\;:k=1,\;\ldots ,\;K\right\}$.

We moreover recommend modeling water CT numbers using the function

(6) $$\begin{array}{l} g\left(\vec{x};\vec{\alpha }\right)=\alpha_{1}+\left(\sum_{i=1}^{5}\alpha_{i+1}x_{i}+\alpha_{i+6}{x_{i}}^{2}\right)\\ +\alpha_{12}x_{1}x_{2}+\alpha_{13}x_{1}x_{3}+\alpha_{14}x_{1}x_{4}+\alpha_{15}x_{1}x_{5}+\alpha_{16}x_{2}x_{3}\\ +\alpha_{17}x_{2}x_{4}+\alpha_{18}x_{2}x_{5}+\alpha_{19}x_{3}x_{4}+\alpha_{20}x_{3}x_{5}+\alpha_{21}x_{4}x_{5} \end{array}$$

and $G_{g}$ to calibrate the set $\{\alpha_{i}\in R:i=1,\;\ldots ,\;21\}.$ The procedure is analogous to the CA models except for the water correction of the data points. Even if water is not chosen as a BM, the water model is created to allow water correction of input images for the decomposition algorithm. This makes CA CE linear in the voxel CT numbers. In particular, if one uses aqueous solutions as BMs, water-filled regions should be evaluated as having zero concentration. Aqueous solutions are a convenient choice for material decompositions in human soft tissue. For a voxel-wise water CT number prediction at arbitrary object size, position, and tube current, we need a water model in each threshold.

If water-filled regions receive a CT number of 0 HU from the CT system, the water model and any water correction is obsolete.

#### 2.1.3. Image Noise Covariance Model Creation

The INCM model H serves for optimally solving Equation (2). For each combination of spectral indices p and q (p,q=1,…, N), the matrix element $H_{pq}$ is the modeled image noise covariance between the p-th and the q-th spectral image. We split the model $H_{pq}$ into

(7) $$H_{pq}=I_{pq}\cdot j_{p}\cdot j_{q}$$

with $I_{pq}$ (p,q=1,…, N) a model of the noise’s correlation between the p-th and the q-th spectral image and $j_{p}$ (p=1,…, N) a model of the noise’s standard deviation in the p-th spectral image. Likewise, we extract correlations and standard deviations from the measured INCMs in $G_{H}$ to acquire ground truth for the models $I_{pq}$ and $j_{p}$. 

We recommend assuming the correlation not to depend on any of the parameters $x_{1}$-$x_{5}$ and thus set $I_{pq}$ to the arithmetic mean of the available ground truth data of $I_{pq}$. We moreover recommend modeling $j_{p}$ using the following function:

(8) $$j_{p}(\vec{x};\vec{\alpha })=\sqrt{\frac{e^{\alpha_{1}+\alpha_{2}\cdot x_{1}}}{x_{2}}}.$$

Because the noise variance ${\left(\sigma_{p}\right)}^{2}$ is proportional to the reciprocal of the tube current $x_{2}$ and increases exponentially with the absorption distance, and thus with the WED $x_{1}$ [40]. See Appendix A for details on how $j_{p}$ was calibrated.

### 2.2. Experimental Setup and Image Acquisition

To apply the method proposed in Section 2.1, we performed two CT measurement series: calibration and test data acquisition. The calibration series provides ground truth data for the model calibration. The test data acquisition serves for inspecting a three-material decomposition.

#### 2.2.1. Material Description

We measured cylindrical WEP phantoms with heights of 100 mm and diameters of 150, 200, 250, 300, and 350 mm (QRM, Möhrendorf, Germany) for the calibration, cf. Figure 2c. The diameters were chosen to represent most clinical applications with different patients and body parts under investigation. These five phantoms were placed on the scanner table with the rotational axis pointing in the axial direction.

**Figure 4:**
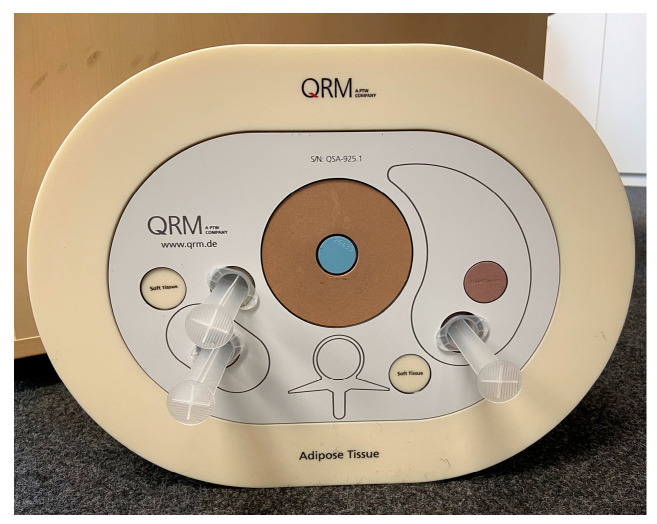
Test scan setup with obese extension ring. In the abdominal phantom, we fill three drill holes, each with one insert: One in the liver-equivalent region, one in the spleen-equivalent region, and one in the soft tissue-equivalent region. The inserts were cyclically swapped between the scans, mentioned in Table 1, to measure each one in all the different regions. The central hole of diameter 100 mm is filled by water-equivalent plastics.

To acquire test data, we measured an abdominal phantom (QRM, Möhrendorf, Germany) with a height of 165 mm and an elliptical cross-section with a width of 300 mm and depth of 200 mm once without and once with an obese extension ring (QRM, Möhrendorf, Germany), cf. Figure 4, of width 400 mm and depth 300 mm (WED = 273 mm and 363 mm). The two configurations represent typical average adult patient sizes for abdominal scans. These two phantoms were positioned on the scanner table with the rotational axis of the central water-equivalent cylinder, visible in Figure 4, pointing in the axial direction.

**Table 1:** CT scan setup for the acquisition of the test data with the phantom visible in Figure 4 for one insert configuration. In total, 360 scans were performed.

Parameter	Value			
Water-Equivalent Phantom Diameter (mm)	273		363	
Tube Current (mA)	375	750	375	750
Repetitions	8	4	32	16
Object Off-centering (mm)	15, 45			

All seven phantoms were positioned as described to achieve axially homogeneous images. Note that all phantoms contained drill holes for placement of syringes.

Our set of M=3 base materials consists of the following:

- Iodine dissolved in water (c=10 mg/mL), hereafter called *iodine*;
- Tungsten dissolved in water (c=10 mg/mL), hereafter called *tungsten*;
- *Water* (c=1000 mg/mL).

By filling 20 mm diameter syringes each with one of these liquids, we prepared three inserts for the phantoms. We chose tungsten to satisfy the requirement of one material clearly interacting with incident X-rays in CT by more than the photoelectric and Compton effect. The concentration of CA in the solutions was chosen to be among the clinically common iodine concentrations below 25 mg/mL [41,42,43].

#### 2.2.2. General Scan Setup

All data were acquired by a dual-source photon-counting scanner (NAEOTOM Alpha, Siemens Healthineers, Forchheim, Germany). This scanner generates up to four energy threshold images, denoted T1, T2, T3, and T4. A threshold image is composed of all photons which deposit energy above the threshold energy in the detector pixel.

We used several fixed scan and reconstruction parameters throughout the experiments as shown in Table 2. In a four-threshold acquisition, the user can perform single-source scans at a tube voltage of 140 kV, rotation time of 0.25 s, and axial collimation of 96 × 0.4 mm. We chose the threshold energy quadruple (20, 55, 72, 90) keV expected to provide the best spectral separation between tungsten, with K-edge energy of 69.5 keV, and the other BMs [44].

The reconstruction algorithm was a weighted filtered backprojection [45]. Neither iterative reconstruction nor post-reconstruction filtering were applied. The chosen slice thickness and increment of 2 mm is used for several examinations of the head and spine, for example [43]. We chose a large field of view to allow retrospective WED calculation.

#### 2.2.3. Calibration

The calibration-specific scan setups are shown in Table 3 and an exemplary phantom in Figure 2c. We performed scans with the listed setups once for each of the three inserts located in the drill hole 30 mm away from the phantom center. We matched the tube currents to the phantom sizes to achieve the same five dose levels for each phantom. After extracting the ground truth for all models as described in the following paragraphs, we applied the recommended procedure described in Section 2.1.2.

To extract ground truth for the material models, we averaged the CT numbers from cylindrical volumes with a diameter of 12 mm over the seven central slices in the reconstructed spectral images. To each of the ROIs, we assigned the distances $x_{3}-x_{5}$ in relation to the circle center.

For the CA models, we averaged the CT numbers in the ROI within the insert of the scans with CA insert as described in Figure 2 and applied the water correction using averaged CT numbers of the corresponding ROI of scans with the water insert. This yields the threshold-wise data $\left\{({\vec{x}}^{k};y_{k})\;:k=1,\;\ldots ,\;K\right\}$ for the LS optimization.

For the water model, we determined the ground truth by averaging the CT numbers from the ROIs in WEP as described in Figure 2.

For the INCM compounds, the ground truth was extracted from the image voxels in the reconstructed images with a distance between 25 and 35 mm to the phantom center and 10 mm to the insert in each threshold image. We did not use all image voxels because a voxel sample with an inhomogeneous CT number background adds low-frequency components to the noise measurement, possibly biasing the measurement [46]. With an incorrect estimated ratio of the noise between the thresholds, the solution of Equation (2) would no longer be the best estimator for the overdetermined LSE in Equation (3) among the linear unbiased ones and would thus suffer from an increased expectation estimation error [47].

#### 2.2.4. Test Data Acquisition

Another measurement series with the parameters described in Table 1 and phantom shown in Figure 4 was performed to test the approach. We varied the phantom off-centering by using different table heights to simulate different levels of non-optimal object positioning on the table.

To investigate systematic errors, we repeated each setup according to Table 1. We matched the repetition numbers to the phantom sizes and tube currents to receive equal noise levels in the evaluation of each setup.

### 2.3. Statistical Analysis

After decomposing the seven central slices of all reconstructed series of the test data into iodine, tungsten, and water using the calibrated models, we analyzed the precision and accuracy of the resulting material maps.

#### 2.3.1. Contrast-to-Noise Ratio

We evaluated the phantom ROIs enclosing inserts for analyzing the CE precision measured by the CNR as follows.

Due to the decomposition process described in Section 2.1, the material map noise was caused by the quantum noise in the threshold images and its propagation into the decomposed images. To understand the influence of the MMD on the material image noise level, we investigated the change in CNR, which typically decreases for material-decomposed images. We defined the input CNR based on the T1 image because it is created by all incident photons on the detector. Thus, the input CNR $a_{\mathrm{in}}$ in one ROI with average CT number quadruple $\vec{y}=(y_{1},\;y_{2},\;y_{3},\;y_{4})$ in one image slice reads

(9) $$a_{\mathrm{in}}=\frac{y_{1}}{\sqrt{\sigma_{\mathrm{in}}}}$$

where $\sigma_{\mathrm{in}}$ is the image noise standard deviation in the T1 image measured in the voxels of the ROI. The output CNR $a_{r,\;\mathrm{out}}$ in the same ROI of the r-th material map with average CE $\rho_{r}$ reads

(10) $$a_{r,\;\mathrm{out}}=\frac{\rho_{r}}{\sqrt{\sigma_{r,\;\mathrm{out}}}}$$

where $\sigma_{r,\;\mathrm{out}}$ is the image noise standard deviation measured analogously to $\sigma_{\mathrm{in}}$. The relative change in CNR, denoted ${\mathrm{CNR}}_{\mathrm{out}}/{\mathrm{CNR}}_{\mathrm{in}}$, of the r-th material was then defined as the ratio of the output and r-independent input CNR:

(11) $${CNR}_{out}/{CNR}_{in}=\frac{a_{r,\;\mathrm{out}}}{a_{\mathrm{in}}}.$$

Due to vanishing input CNR in CA-free ROIs and output CNR close to zero in ROIs with one CA in the map of a different BM, ${\mathrm{CNR}}_{\mathrm{out}}/{\mathrm{CNR}}_{\mathrm{in}}$ according to Equation (11) is a meaningful number and was computed uniquely in the following cases:

- Iodine CE in the iodine insert;
- Tungsten CE in the tungsten insert.

We finished by taking the arithmetic mean over the central slices and possible scan repetitions to mitigate the contribution of quantum noise to the CNR quotient. Note that due to varying photon statistics, the noise in $a_{\mathrm{in}}$ and $a_{r,\mathrm{out}}$ changes with the tube current. As the material decomposition is a linear operation on the input image, cf. Equation (2), we do not expect a dependence of ${\mathrm{CNR}}_{\mathrm{out}}/{\mathrm{CNR}}_{\mathrm{in}}$ in the tube current.

#### 2.3.2. Estimation Accuracy

To investigate the accuracy of the concentrations resulting from the MMD, we compared them with the true concentrations. We expected a water concentration of 1000 mg/mL throughout the phantom. We expected an iodine concentration of 10 mg/mL in the iodine insert and 0 mg/mL otherwise. We expected a tungsten concentration of 10 mg/mL in the tungsten insert and 0 mg/mL otherwise.

We averaged the CT numbers within each of the three inserts. The resulting mean values of the seven slices in all repetitions were then averaged to mitigate the contribution of quantum noise to the CE errors. We then took the difference between the averaged means and the expected value.

## 3. Results

**Figure 5:**
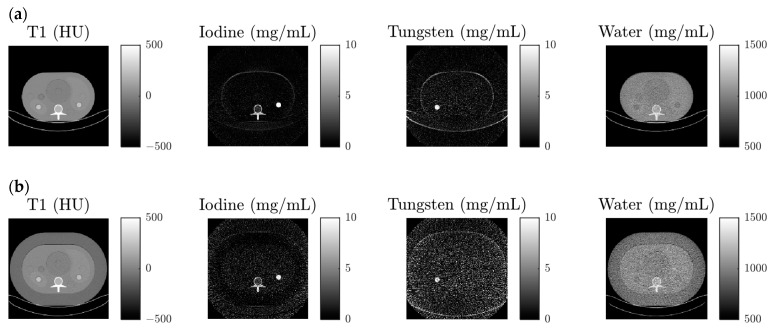
Material decomposition input (T1 image) and output (material maps with contrast agent inserts visible as bright patch in the respective map) of the test data configuration with tube current 750 mA, 15 mm off-centering, tungsten in the left and iodine in the right insert (**a**) without obese extension ring and (**b**) with the obese extension ring. Peripheral image regions were cropped.

Exemplary material maps from two test data setups are visualized in Figure 5. The CA maps mainly show the insert containing the respective material. For each phantom, the tungsten map is noisier than the iodine map. The maps show the WEP and the water insert with a concentration of approximately 1000 mg/mL, lower concentration in the obese extension ring, and higher concentration in the CA inserts and abdominal material. The material maps for the phantom with extension ring are noisier than the ones for the phantom without.

### 3.1. Contrast-to-Noise Ratio

**Figure 6:**
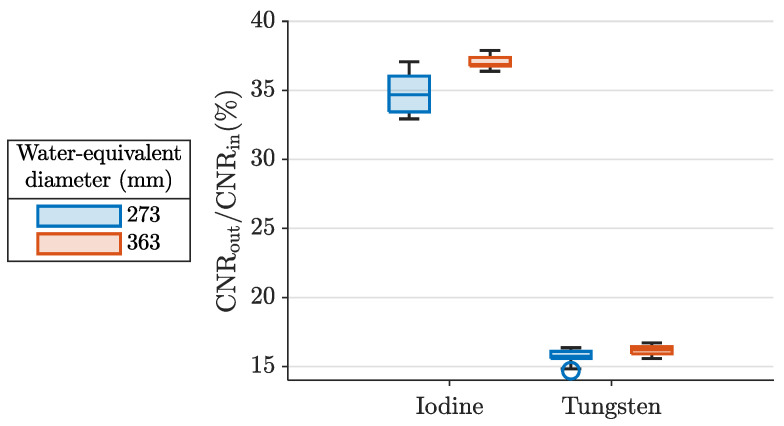
${\mathrm{CNR}}_{\mathrm{out}}/{\mathrm{CNR}}_{\mathrm{in}}$ between each contrast agent map and the T1 image, of the scans listed in Table 1. Each box displays the median of all contributing data points and is bounded by the lower and upper quartiles. The whiskers extend to the extreme values except for outliers, which are defined by being more than 1.5 interquartile ranges away from the top or bottom of the box.

Figure 6 shows ${\mathrm{CNR}}_{\mathrm{out}}/{\mathrm{CNR}}_{\mathrm{in}}$ for each CA. We found ${\mathrm{CNR}}_{\mathrm{out}}/{\mathrm{CNR}}_{\mathrm{in}}$ in the iodine map within the range of ±3% around the arithmetic mean at 36% and in the tungsten map in the range of ±1% around the arithmetic mean at 16%. For each CA, the values tend to be higher for the larger phantom diameter.

### 3.2. Estimation Accuracy

**Figure 7:**
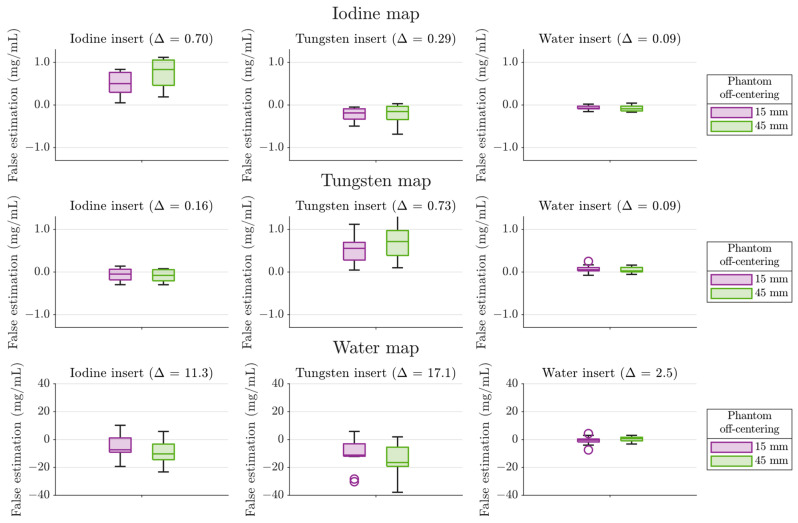
Errors in the estimated material concentrations of the test data using the models described in Section 2.1. There is one column for each of the inserts. The box construction is analogous to Figure 6. Positive errors indicate an overestimated concentration, while negative errors indicate an underestimation. Δ is the root mean square of all false estimations in units of mg/mL.

Figure 7 shows the deviations of the CEs from the true values for the nine combinations of material maps (iodine, tungsten, and water) and inserts (iodine, tungsten, and water). We found average false estimates of 0.4 mg/mL in the iodine and in the tungsten map and 11.9 mg/mL in the water map. This means an average error of 4% in the CA concentration with respect to the insert concentration of 10 mg/mL and 1% in the water map with respect to the total concentration of 1000 mg/mL. Most of the false estimates in the CA inserts except for the tungsten map–iodine insert case were due to systematic effects, viewing propagated quantum noise of 0.01 mg/mL, 0.03 mg/mL, and 0.9 mg/mL on each data point in the iodine map, the tungsten map, and the water map, respectively.

The CE in the water insert, with maximum errors below 2% with respect to the total concentration, was more accurate than in the CA inserts. In the iodine insert, the iodine concentration was overestimated with medians of 5 and 8% for low and high phantom off-centering, respectively. Meanwhile, the water concentrations were underestimated with a median of at most 1% independent of the phantom position. In the tungsten insert, the tungsten concentration was overestimated with medians of 6% and 7% for low and high phantom off-centering, respectively. Meanwhile, the iodine and water concentrations were underestimated with a median of 2% or less independent of the phantom position. The entirety of false estimates in the CA map–CA insert pairs tended to be overestimated. 

For each map–insert pair, the 15 mm off-centered phantoms showed more accurate results than the 45 mm off-centered phantoms. The central quartiles did not show systematically higher false estimations.

We would like a conservative upper boundary for systematic CA CE errors at arbitrary CA concentration c. As a voxel’s absorption equals the sum of the absorptions of all included materials in the thin-absorber limit and as the CE is linear in the voxel’s CT number quadruple, the largest errors measured at c=0 (pure water) and c=10 mg/mL, denoted as $\varepsilon_{0}$ and $\varepsilon_{1}$, implied the following upper boundary:

(12) $$\varepsilon_{0}+\frac{\varepsilon_{1}}{10\;\mathrm{mg}/\mathrm{mL}}\cdot c.$$

For the phantom setup used during the test data acquisition, we expected the iodine and tungsten CE in a voxel of CA concentration c for a well-positioned object ($x_{5}=15\;\mathrm{mm})$ to suffer from a systematic inaccuracy of less than 0.2 mg/mL+0.11·c.

## 4. Discussion

### 4.1. Contrast-to-Noise Ratio

For each CA, ${\mathrm{CNR}}_{\mathrm{out}}/{\mathrm{CNR}}_{\mathrm{in}}$ varied by only a few percent across the different configurations. On average, the CNR declined to 36% and 16% for iodine and tungsten maps, respectively.

The stronger CNR reduction for tungsten and thus higher noise in the tungsten map compared to iodine, visible in Figure 5, is not surprising: Iodine’s absorption curve, on the one hand, strictly decreases in the diagnostic energy range and leads to monotonously decreasing CT numbers with increasing threshold energy, which separates well from water whose CT number is assumed to be identical in all thresholds. Tungsten’s absorption curve, on the other hand, contains a K-edge, which leads to a relatively weak variation of the threshold CT numbers and consequently to a worse separation from water.

We attribute the trend to a weaker CNR reduction for larger objects to stronger beam hardening and thus better spectral resolution of the system. However, for larger objects irradiated with equal X-ray tube flux, the noise in spectral input images is higher due to a lower photon flux on the detector. So, despite a slightly weaker CNR reduction, the material map is noisier for larger objects as can be seen in Figure 5.

### 4.2. Estimation Accuracy

Figure 7 shows mainly systematic CE errors resulting from applying the created models for a MMD of the test data into iodine, tungsten, and water. Lower false estimates in the case of low phantom off-centering support the best practice guideline to adjust patients as close to the isocenter as possible. As most absolute errors are on the same level as in the low off-centering case for all map–insert pairs, our approach shows certain robustness against object positioning.

We explain the observed tendency to overestimate CA concentrations in the CA inserts in the corresponding inserts by the ignored parameter $x_{4}$ in the CA models. We used $x_{4}=30$ mm throughout the calibration scans but used larger values of $x_{4}$ for the test data acquisition. Beam hardening induces a higher CT number for CA closer to the object boundary resulting in higher CEs.

We attribute the water concentration underestimation in the CA inserts and the underestimation in the tungsten map–iodine insert case to the surrounding materials equivalent to soft tissue, liver, spleen, and spinal column. Their CT number decreases with increasing threshold energy. Given $x_{1}$, based on the CT numbers in T1, we thus expect a higher photon flux through the CA insert in the upper thresholds T2, T3, and T4 in the abdominal phantom than in a phantom of water-equivalent material with equal $x_{1}$. This surplus is larger with larger photon energy.

The higher flux passing through the CA inserts decreases the CT numbers in the upper thresholds. This is compensated by an increased CA CE and a decreased water CE both for the iodine and the tungsten insert. The tungsten insert requires considering the K-edge which leads to a higher CT number in T3 than in T2 and a lower CT number in T4 than in T2. The increasing photon surplus with increasing threshold energy enlarges the CT number difference between T3 and T4 more than between T2 and T3. A negative iodine concentration compensates for this relatively lower difference between T2 and T3. 

Thus, for targets whose absorption is not dominated by water, the WED is no longer a sufficient parameter to describe the pre-attenuation for an image voxel of interest.

A further increase in accuracy could be achieved by adding more parameters to the models. However, this would increase the number of required calibration scans, thus increasing the time needed to calibrate each material and hence limiting the method’s practicability. 

We assume the achieved estimation accuracy and CNR to be sufficient for applications aiming to differentiate image regions with different content, such as coronary CT angiography (CCTA) or stent imaging which have already been tested with tungsten-based CA [20,22]. According to the results in Section 3.2, iodine and tungsten with a concentration of 3 mg/mL or above are detectable without a problem, despite systematic errors resulting from MMD into iodine, tungsten, and water. According to the results in Section 3.1, we predict the exemplary contribution of quantum noise to the CA CE in a volume of 16 mL, like a medium plaque [48], scanned in a CCTA with exposure 255 mAs [49]. In a patient with a medium WED of 300 mm, the standard deviation on the CE in the averaged volume equals 0.3 mg/mL and 0.7 mg/mL for iodine and tungsten. This makes the medium plaque easy to distinguish from surrounding iodine- or tungsten-based CA.

Finally, Equation (12) may not be valid for arbitrarily high insert concentrations in the examined target due to a broken thin-absorber limit and thus non-proportional CT number and insert concentration, particularly if photon starvation reduces the incident signal below the detector sensitivity.

## 5. Conclusions

We presented a method for performing a MMD based on multiple energy-threshold input images from an energy-discriminating PCD CT system. The calibration does not require manually fine-tuned initial conditions. No prior knowledge is required to apply the calibrated models. All model computations are analytical, resulting in a fast and robust algorithm execution. It was designed for anthropomorphic geometries at arbitrary table heights and exposures.

We applied our approach to a commercial PCD scanner using a set of three BMs with suitable properties: Iodine, tungsten, and water. We evaluated its accuracy for several phantom configurations and scan parameters representative of typical clinical protocols and examined body parts. The investigation of more complex setups as a study involving biological specimens is planned as follow-up of this work.

We used a non-iterative image reconstruction to be as close to the statistical reality as possible. In clinical practice, an iterative reconstruction with lower quantum noise per image voxel and thus lower contribution of quantum noise to CE on each material map voxel is usually used [50].

The measured objects were axially homogeneous, whereas some clinical applications with axial inhomogeneity require a smaller slice thickness than what was used in this work. We scanned 20 mm diameter syringes with CA and water and evaluated volumes of 1.6 mL in the syringes to assess the algorithm-inherent performance. We expect the results for the algorithm’s precision and accuracy to be valid also for smaller objects. Investigating very small objects such as narrow vessels with sizes close to the CT system’s spatial resolution will introduce additional errors due to the partial volume effect.

As the images acquired to test the method was used to optimize the model types, both datasets are not entirely independent. The method should thus be tested with additional measurements in different setups for a more reliable validation.

The chosen threshold energy quadruple provides the best spectral separation between tungsten and the other BMs. Using another predefined threshold energy quadruple, we expect a stronger CNR decline in the material images and larger CE errors. Further studies should also investigate the reliability of the presented approach when introducing a BM with less optimal K-edge regarding the spectral bins or thresholds.

The method is not limited to a three-material decomposition. As the used system provides four spectral datasets per scan, a four-material decomposition is possible. Upcoming PCD CT scanners will likely be able to use more than four spectral datasets per scan. Decomposition into as many materials as there are spectral datasets is then possible. The quality of such MMDs would be highly dependent on the specific choice of BMs and scan parameters, especially on the optimal choice of spectral bins or thresholds.

## Figures and Tables

**Table 2:** Fixed parameters for the scans.

Parameter	Value
Tube Voltage (kV)	140 (Tube A only)
Threshold Energies (keV)	20, 55, 72, 90
Scan Mode	Spiral
Rotation Time (s)	0.25
Pitch	0.55
Axial Collimation (mm)	96 × 0.4
Reconstruction Algorithm	Weighted Filtered Backprojection
Kernel	Qr40
Field of View (mm)	500
Matrix Size	512 × 512
Slice Thickness (mm)	2.0
Slice Increment (mm)	2.0

**Table 3:** CT scan setup for the calibration measurements of each base material with phantom configuration shown in Figure 2. For each base material, 75 scans were performed in total.

Parameter	Value				
Phantom Diameter (mm)	150	200	250	300	350
Tube Current (mA)	60, 80, 100, 120, 140	110, 140, 180, 210, 250	170, 220, 280, 330, 390	240, 320, 400, 480, 560	330, 440, 540, 650, 760
Object Off-centering (mm)	0, 30, 60				
Insert Distance to the Object Center (mm)	30				
Resulting Insert Distances to the Isocenter (mm)	0, 42, 67				

## Data Availability

The dataset is available on reasonable request from the authors.

## Appendix A

In the following paragraphs, we discuss the model functions indicated in Section 2.1.2 and Section 2.1.3 in more detail.

Our water model function indicated by Equation (6) is a linear combination of polynomials in the parameters $x_{1}$ − $x_{5}$ as the most generic choice. We found the polynomials up to quadratic order to be the best trade-off between accuracy and robustness after also evaluating both the analogous model without quadratic terms and the model extended with cubic terms with the test data. Either case showed larger errors in material CEs from the respectively derived material maps.

Regarding the CA model function indicated by Equation (4), the iodine CT number dependencies as shown in Figure 3 suggest using more complex polynomials than constants and linear ones in the parameters $x_{1}-x_{3}$. We generated models each for the affine-linear case, the quadratic case, and with cubic terms in $x_{1}{-x}_{3}$, applied them to the test data and found the lowest material CE errors in the quadratic case. For this reason, we chose the linear combination of polynomials up to quadratic order in $x_{1}-x_{3}$.

We did not use other parameters other than $x_{1}-x_{3}$ for the CA models to avoid multiplying the workload because we aimed for a practical workflow. Adding the voxel distance to the object center $\left(x_{4}\right)$ to the set of model parameters by calibrating multiple CA inserts together in one phantom is not recommended to avoid beam hardening effects between the inserts. For the water model, however, we were able to use the parameters $x_{1}$ − $x_{5}$ at the same workload as for each CA, because the phantoms made of WEP allowed for extracting ground truth CT numbers at positions in the phantom for each scan.

We did not use $x_{4}$ or the phantom off-centering $x_{5}$ instead of the distance to the isocenter $\left(x_{3}\right)$ for the following reasons: First, using $x_{4}$, which one may calibrate by changing the insert position within an isocentered phantom, instead of $x_{3}$ would give more accurate results for isocentered objects. However, using $x_{3}$ provides more robustness against object off-centering. Second, the phantom off-centering ($x_{5}$) plays a minor role compared to the other parameters, since for fixed coordinates $x_{1}-x_{4}$ the relative impact of varying $x_{5}$ in the range between 0 and 40 mm on the iodine CT number is less than 1% at 10 mg/mL.

Regarding the INCM, we are left with the algorithm to calibrate $j_{p}$ (p=1,…, N) indicated in Equation (8). To apply LS methods to the non-linear model $j_{p}$, the user can proceed as follows:

First, among the ground truth values of $j_{p}$, those acquired at the same $x_{1}$ and $x_{2}$ are averaged using the quadratic mean, which leads a new reference data set $\left\{\left(x_{1}^{k},x_{2}^{k};\sigma_{k}\right):k=1,\;\ldots ,K\right\}$. Second, the linear function l with

(A1) $$l\left(x_{1};\vec{\alpha }\right)=\alpha_{1}+\alpha_{2}\cdot x_{1}$$

is fitted to the data points $\left\{\left(x_{1}^{k};\tau_{k}\right):k=1,\;\ldots ,K\right\}$ with $\tau_{k}=\log\left[{\left(\sigma_{k}\right)}^{2}\right]+\log x_{2}^{k}$ for all k=1, …,K, using ordinary LS. The resulting optimized model parameters $\alpha_{1}$ and $\alpha_{2}$ coincide with the model parameters of the desired function $j_{a}$: After taking the exponential of both sides of $\tau_{k}=\alpha_{1}+\alpha_{2}\cdot x_{1}^{k}$, dividing by $x_{2}^{k}$, and taking the square root of both sides, the user ends up with

(A2) $$\sigma_{k}=\sqrt{\frac{e^{\alpha_{1}+\alpha_{2}\cdot x_{1}^{k}}}{x_{2}^{k}}}$$

in accordance with Equation (8) and ground truth $\left\{\left(x_{1}^{k},x_{2}^{k};\sigma_{k}\right):k=1,\;\ldots ,K\right\}$.

Our INCM model H, calibrated by ground truth as described in Section 2.2.3, did not consider $x_{1}-x_{5}$ for $I_{pq}$ (p,q=1,…, N) and $x_{3}-x_{5}$ for $j_{p}\;(p=1,\ldots ,\;N)$ and ignored the possibly altered noise in CA-filled inserts. To assess the consequence on the material decomposition, we worked on the test data as follows:

1. For each scan and each ROI, we measured the INCM and averaged the matrices acquired in all reconstructed slices and all repetitions of equal setup. Assigning the ROI center coordinates $x_{3}$ and $x_{4}$ to each ROI, we created a look-up table for the INCM for each material and ROI in the parameters $x_{1}-x_{5}$.
2. We performed an iodine-tungsten-water-decomposition for each scan and ROI based on our material models once using the matching INCM from the look-up table and once using H.
3. We averaged the resulting material concentrations ROI-wise over all slices and scan repetitions. We then took the absolute differences between the resulting concentrations of the two decompositions. Those did not exceed 0.1 mg/mL in the CA maps and 2 mg/mL in the water map.

Because of these findings, we decided not to recommend a more complex INCM model.
